# DRUJ arthroscopy

**DOI:** 10.1186/1753-6561-9-S3-A32

**Published:** 2015-05-19

**Authors:** Toshiyasu Nakamura

**Affiliations:** 1 Department of Orthopaedic Surgery, Sanno Hospital, 107-0052, Tokyo, Japan

## 

DRUJ arthroscopy was rarely reported compared with radiocarpal or midcarpal joint arthroscopy. We describe our technique, and findings of DRUJ scope for DRUJ instability cases.

Technique: The portal was made at a very distal area of the DRUJ, just proximal of the TFCC surface. The 1.9 mm oblique view arthroscope was used. Curved blunt mosquito forceps were firstly inserted to the DRUJ through this portal and the arthroscope was then introduced into the joint. The ulnar head, sigmoid notch of the radius, proximal surface of the TFCC, radioulnar ligament (RUL) origin at the fovea, DRUJ capsule were observed via DRUJ arthroscopy. To check the condition including tension and tear area of the RUL, a 23G needle was used.

Patients: Since 2000, we performed DRUJ arthroscopy in 196 wrists of 194 cases. There were 108 male and 86 female, with an average age of 32 (range, 15 to 63). Right wrists were 109, left 85, and one bilateral. All cases demonstrated moderate to severe DRUJ instability.

Results: The DRUJ surface including the ulnar head and sigmoid notch of the radius could be observed in all cases. We could observe the RUL origin at the fovea in 170 wrists, while no visualization at the fovea was obtained in 26 wrists due to severe proliferation of the synovial at the fovea. There was absence of the RUL with/without scaring in 58, the partial avulsion of dorsal portion of the RUL in 35, the partial avulsion of the palmar portion of the RUL in 9, relaxed RUL in 32, fibrillation on the RUL surface in 20, and a normal RUL in 16 (may be related to the horizontal type tear of the TFCC). We performed synovectomy if synovial proliferation and fibrillation was present, open repair/reconstruction of the TFCC in cases with a scarred, empty RUL and in complete disruption, and ulnar shortening in cases with a relaxed RUL.

Conclusion: DRUJ arthroscopy is useful especially for evaluation of the RUL condition and selection of treatment.

